# Effects of a Digital Functional Exercise Program on the Disease Activities and Physical Capabilities of Patients With Ankylosing Spondylitis: Protocol for a Randomized Controlled Trial

**DOI:** 10.2196/67556

**Published:** 2025-07-07

**Authors:** Xingkang Liu, Xiaojian Ji, Lidong Hu, Tianyu Jiang, Ang Min, Lulu Zeng, Yiwen Wang, Meihui Zhao, Jian Zhu, Feng Huang, Changshui Weng, Zheng Zhao

**Affiliations:** 1 Department of Rehabilitation Medicine The Second Medical Center Chinese PLA General Hospital Beijing China; 2 Department of Rheumatology and Immunology The First Medical Center Chinese PLA General Hospital Beijing China; 3 Jiakang Zhongzhi Technology Company Beijing China

**Keywords:** ankylosing spondylitis, digital health, Digital Functional Exercise Program, physical capabilities, physical exercise, randomized controlled trial

## Abstract

**Background:**

Ankylosing spondylitis (AS), a chronic inflammatory disease, causes spinal stiffness, functional impairment, and reduced quality of life. While exercise is critical for managing AS, traditional home-based programs lack real-time supervision to ensure movement quality and adherence. Emerging digital tools like inertial sensors may address this gap, but their clinical impact remains unproven.

**Objective:**

This study aims to evaluate the feasibility and efficacy of a Digital Functional Exercise Program (DFEP) using wearable sensors and real-time feedback for patients with AS.

**Methods:**

This single-blind randomized controlled trial (ChiCTR2300068327) enrolled 80 adults with AS from the Chinese People’s Liberation Army (PLA) General Hospital. Participants were randomized 1:1 to: DFEP Group: 24-week sensor-guided exercises via the Healbone Mini Program (Jiakangzhongzhi, Co), with real-time feedback and remote physiotherapist oversight. Control Group: standard home-based exercises with written instructions. The primary outcome is the change in Ankylosing Spondylitis Disease Activity Score with C-reactive protein (ASDAS-CRP) at 24 weeks. Secondary outcomes include Bath Ankylosing Spondylitis Disease Activity Index (BASDAI), Bath Ankylosing Spondylitis Functional Index (BASFI), Bath Ankylosing Spondylitis Metrology Index (BASMI), Assessment of Spondyloarthritis International Society Health Index (ASAS HI), 36-Item Short Form Survey (SF-36), pain visual analog scale, Five-Times-Sit-to-Stand, 4-meter walk test, Hamilton Anxiety Scale (HAMA), Hamilton Depression Scale (HAMD), and adherence. Analyses will follow an intention-to-treat approach with the Last Observation Carried Forward for missing data; continuous variables will be compared with *t* tests or ANOVA, and categorical variables with chi-square tests, using a 2-sided =.05.

**Results:**

We have screened 216 outpatients who may be eligible subjects, among them 126 outpatients who initially met the inclusion criteria. After evaluating clinicians performed face-to-face assessments, 23 lacked a stable medication regimen, 5 presented with severe cervical vertebral bridges, 3 reported regular structured exercise, 2 could not be reached after prescreening, 10 had significant cardiovascular disease, and 3 declined participation due to time constraints. Ultimately, 80 eligible participants were enrolled, with 36 randomly allocated to the intervention group and 44 to the control group. Recruitment (February 2023-March 2024) and follow-up (concluding September 2024) are complete. Data analysis (November 2024) and result dissemination (April 2025) are pending.

**Conclusions:**

This trial is the first to test a fully digital, sensor-based exercise program for AS. If effective, DFEP could offer a scalable, cost-effective solution for home rehabilitation in AS and related conditions.

**Trial Registration:**

Chinese Clinical Trial Registry ChiCTR2300068327; https://www.chictr.org.cn/showproj.html?proj=190897

**International Registered Report Identifier (IRRID):**

DERR1-10.2196/67556

## Introduction

Ankylosing spondylitis (AS) is a chronic, systemic rheumatic disease that serves as the prototype of spondyloarthritis and is primarily characterized by inflammation of the spine [1]. The principal clinical features of AS include reduced physical activity, pain, stiffness, sleep disturbances, decreased spinal mobility, and psychological consequences such as depression [2]. Less frequently, AS affects peripheral joints and causes extra-articular manifestations, such as Achilles tendinitis, uveitis, psoriasis, and inflammatory bowel disease.

The main objectives of treatment are to alleviate symptoms, improve functioning, maintain the ability to work, decrease disease complications, and minimize skeletal damage. However, pharmacological therapy alone is often insufficient in managing AS, especially in patients with existing functional limitations. The importance of exercise in AS has been emphasized in the Assessment of SpondyloArthritis international Society/European League Against Rheumatism (ASAS/EULAR) recommendations for the management of AS [3]. Although there is no clear consensus on which specific exercise is the most effective, a comprehensive functional exercise program including stretching, mobilization, strengthening, and balance exercise is recommended.

A recent meta-analysis found that both supervised and home-based exercise programs are beneficial in reducing Bath Ankylosing Spondylitis Metrology Index (BASMI), Bath Ankylosing Spondylitis Disease Activity Index (BASDAI), and Bath Ankylosing Spondylitis Functional Index (BASFI) in patients with AS, with supervised exercise programs showing greater efficacy than home-based exercise programs in decreasing disease activity [4]. The main reason for this result is that supervised exercise could improve the compliance of patients with exercise, improve the accuracy of exercise, leading to better therapeutic outcomes. Fortunately, the development of electronic medicine is being driven by mobile technologies, including smartphones and other wireless communication devices [5]. In recent years, mobile devices have experienced dramatic innovations, enabling anytime, anywhere, and real-time connectivity and interactions. Unlike single physiological indicators such as heart rate, functional exercise involves complex multijoint movements, dynamic ranges of motion, and postural corrections. Traditional home-based exercise models lack real-time feedback, making it challenging to simultaneously evaluate movement accuracy, adherence, and clinical progress. While digital health technologies have advanced significantly, few studies have rigorously tested scalable solutions that merge the flexibility of home-based routines with the precision of supervised guidance—a critical gap hindering the development of accessible, long-term management strategies for AS. Building on our prior work with a WeChat mini-program to monitor home-based functional exercises in patients with AS [6], we have now integrated inertial sensors into an upgraded WeChat applet. This innovation enables real-time tracking of movement quality and adherence, addressing the limitations of traditional models by providing objective, quantifiable feedback akin to in-person supervision.

This randomized controlled trial aims to evaluate the feasibility and efficacy of a digital functional exercise program for patients with AS. We hypothesize that the intervention group using the digital platform (Healbone) will demonstrate superior improvements in disease activity, measured by the Ankylosing Spondylitis Disease Activity Score with C-reactive protein (ASDAS-CRP), compared to the control group receiving standard home-based exercises. Secondary hypotheses posit greater enhancements in physical function (BASFI), spinal mobility (BASMI), and quality of life (36-Item Short Form Survey [SF-36]) in the intervention group, attributable to improved exercise adherence and real-time feedback mechanisms.

## Methods

### Study Design

This randomized, prospective, controlled study was a single-blind trial, conducted at the Department of Rheumatology and Immunology, Chinese People’s Liberation Army (PLA) General Hospital. After baseline assessments by an assessor who was blinded to the group assignments, eligible participants were randomly assigned to the trial group or the control group after providing informed consent. Assessors were blinded to group allocation, but participants were aware of their assigned intervention (digital platform or manual-based exercise) due to the inherent nature of the interventions. This pragmatic design reflects real-world conditions where patients would typically know their treatment modality. This protocol is in line with CONSORT (Consolidated Standards of Reporting Trials) guidelines (checklist in Multimedia Appendix 1).

### Participants

Patients diagnosed with AS according to the 1984 modified New York criteria [7] at the Chinese PLA General Hospital, either as a first-time diagnosis or previously, were enrolled in the study and assigned to 2 groups. In addition to collecting their demographic characteristics (age, gender, weight, height, and BMI), the patients were also questioned about occupation, main symptoms, time of diagnosis, and drug usage (nonsteroidal anti-inflammatory drug, disease-modifying antirheumatic drugs, including biologics). Other inclusion criteria were aged ≥18 years, stable drug treatment in the preceding month, and ASDAS-CRP between 1.3 and 3.5 [8]. Exclusion criteria were cardiovascular disease or clinical status at high risk, screened with the American Heart Association/ACSM Health/Fitness Facility Preparticipation Screening Questionnaire [9], cervical vertebra bridges, surgery within the preceding 6 months, regular exercise in the preceding 3 months (eg, yoga, Tai Chi, Baduanjin 3 or more times per week, 20 minutes per time), and factors leading to the inability to receive regular exercise rehabilitation (such as language impairment, difficulty in understanding, and limited movements).

### Procedures

All subjects in this study were patients diagnosed with AS in the outpatient Department of Rheumatology and Immunology, in the Chinese PLA General Hospital. First, the potentially eligible subjects were screened by a study physician, and the exclusion criteria of all screened patients were recorded. Then, the informed consent form was signed for eligible subjects, and the baseline data were collected in detail. The trial implemented evaluation physician blinding at both baseline and follow-up phases, with group allocation data concealed via an opaque envelope. Postassessment allocation to a physical therapist for exercise prescription occurred through a separate workflow managed by the trial coordinator. Finally, a personalized exercise rehabilitation scheme was given to all the subjects by the physical therapist according to the patient’s function evaluation (Multimedia Appendix 2).

### Interventions

A 24-week tailored functional exercise program, consisting of stretching, mobilization, and strengthening exercises, was designed based on the patients’ basic functional abilities following randomization. All patients had a 1-hour training session at entry, providing information about AS and the purpose of physical exercises.

**Figure 1:**
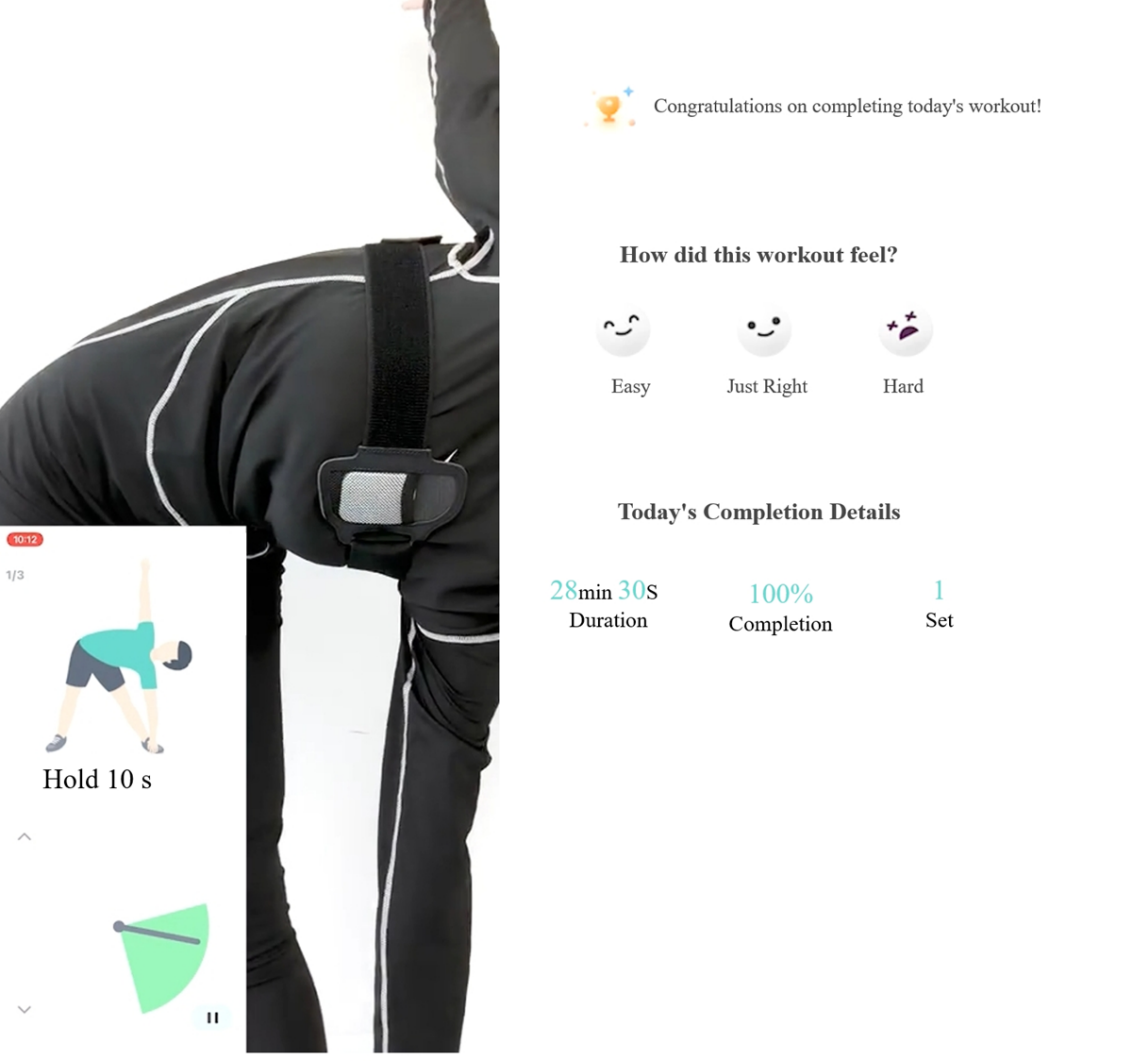
Schematic representation of training feedback.

In addition to a face-to-face session, participants in the experimental group received a digital exercise program given by the physiotherapist. Participants in the intervention group were taught the usage of the digital exercise project that will be delivered via WeChat applets to individual exercise interfaces. In the first step, patients needed to wear a sensor on their chest, close to their armpits, and then connect to their mobile phones via Bluetooth. After a successful connection, they carried out the personalized workout program listed in Multimedia Appendix 2. During exercise, participants in the experimental group received real-time feedback on movement standardization and joint angles via the WeChat-based exercise applets. Postexercise feedback was systematically collected using the Borg Rating of Perceived Exertion scale (Figure 1), enabling adjustments to exercise regimens based on participants’ subjective experiences of effort.

The physiotherapist’s management platform enabled remote monitoring of participants’ home exercise compliance through real-time data transmission from wearable sensors. Participants were instructed to complete exercises 3-4 times per week, with session frequency and duration tailored to their baseline functional assessments. To reinforce adherence, automated reminders were triggered via the platform if a participant missed exercise sessions for 7 consecutive days, prompting re-engagement through personalized notifications (eg, motivational messages and corrective feedback). Physiotherapists reviewed compliance metrics weekly to adjust exercise prescriptions or provide additional guidance as needed.

The participants in the control group received a home-based exercise program, and an exercise manual was given to each participant. The participants in the control group were also recommended to perform the exercise 3-4 times per week. Participants in the control group were provided with a paper logbook to record exercise frequency, duration, and perceived exertion. Research assistants conducted biweekly phone follow-ups to collect logbook data, reinforce adherence, and address participant questions. Adherence was calculated as the self-reported ratio of completed sessions to prescribed sessions (3-4 sessions/wk), mirroring the intervention group’s digitally recorded adherence metric.

To minimize deviation risks, we established WeChat patient management groups for each participant (both control and intervention groups) comprising: the enrolled patient, a rheumatologist, a physical therapist, and a digital health assistant. The physical therapist provided targeted assistance in the intervention group under two predefined conditions: (1) when abnormal exercise difficulty was detected through our background monitoring system, and (2) if the intervention group participants failed to complete exercises consecutively for 1 week.

For participants in the control group, physical therapists solely addressed inquiries related to medical conditions and exercise clarification, without providing proactive rehabilitation guidance or progress monitoring. All communication occurred digitally via the WeChat platform except during scheduled in-person outpatient follow-up visit. No additional face-to-face meetings were conducted beyond these standard clinical appointments.

### The Digital Platform

The digital exercise platform, Healbone, was developed through a participatory design process involving patients with AS and their relatives, rheumatology specialists, physical therapists, and a technical team. Healbone serves as a shared communication platform accessible to patients, their families, and associated health care professionals. Patients must provide written consent regarding data sharing. All information is uniformly available to all participants. Health care personnel and researchers have access to additional tools for managing care, obtaining overviews of patient conditions, and simultaneously monitoring multiple patients. The platform also functions as a coordination and communication tool among patients, relatives, and health care professionals.

All measurements are securely stored in a database. The web-based Healbone platform includes multiple features designed and developed through user-driven innovation. Facilities and input data specific to individual patients, such as symptom scores, are accessible and available to authorized personnel. The main features include:

1. Video consultation: Patients can conduct video calls with the health care team to receive professional advice and support.
2. Messaging: The platform facilitates message exchange between patients and doctors, enhancing communication and follow-up.
3. Personalized exercise plans: Tailored exercise plans are generated based on the patient's specific condition, with daily reminders.
4. Exercise tracking: Real-time monitoring and recording of patients' exercise activities.
5. Educational resources: The platform provides educational materials on AS management and exercise, aiding patients in better understanding and managing their condition.
6. Calendar: Patients can record appointments and other activities with the health care team for easy viewing.
7. Status tracking: Overview of questionnaire results completed by patients, available for viewing by patients and their authorized relatives and health care professionals.
8. To ensure the security of patient data, Healbone uses the following measures: (1) data encryption: all data are encrypted during transmission and storage; (2) user authentication: two-factor authentication ensures that only authorized users can access the platform; and (3) privacy policy: The platform complies with General Data Protection Regulation (GDPR) and other relevant data privacy regulations to protect user privacy.

**Figure 2:**
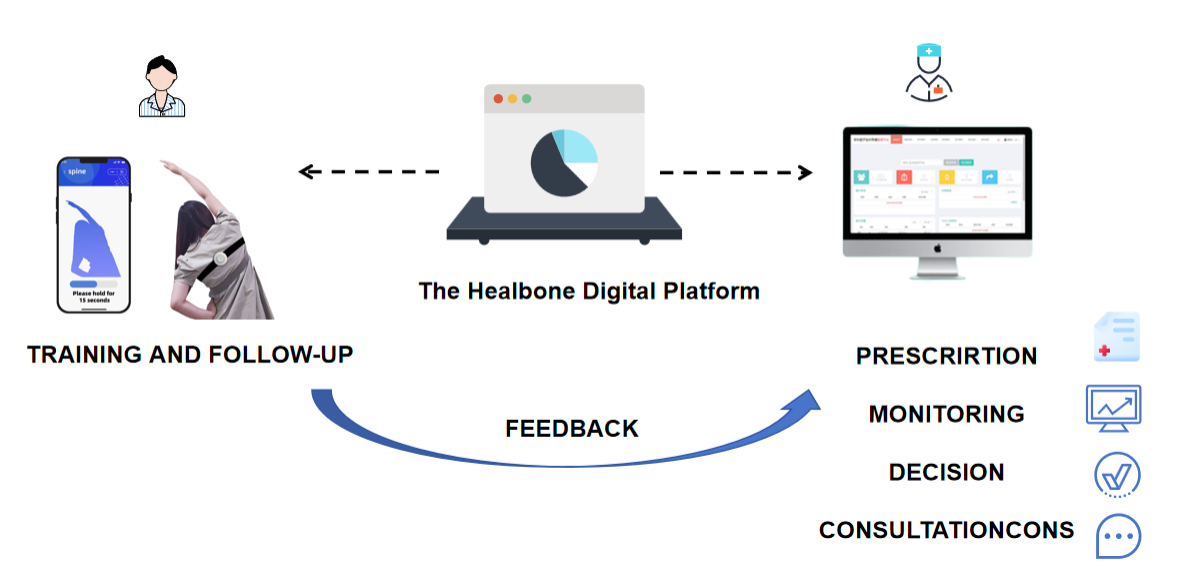
The overview diagram of the Healbone digital platform.

In addition, Healbone can integrate with electronic health record systems and other medical devices, enabling comprehensive data management and analysis to provide holistic health management support for patients. Through these features, the platform not only improves patients’ physical abilities and quality of life but also equips clinicians with effective tools for enhanced monitoring and treatment plan adjustments (Figure 2).

**Figure 3:**
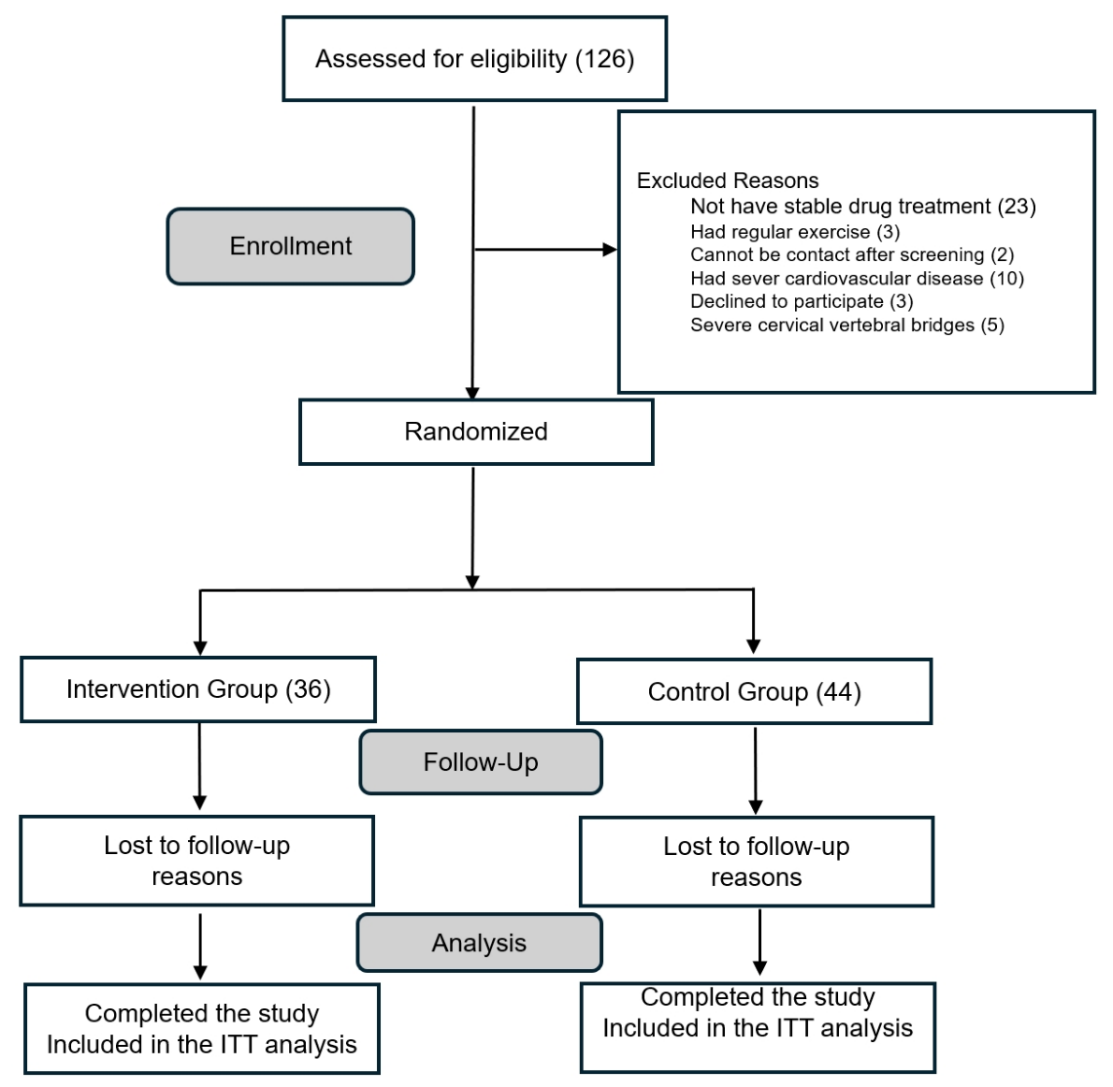
Treatment assignments and withdrawal in the participants. ITT: intention-to-treat.

### Randomization Scheme

All patients were recruited by a physician and randomly assigned to either the intervention or control group in a 1:1 ratio. To ensure allocation concealment, a computer-generated randomized table of numbers was prepared by an independent individual before the study’s commencement. The numbered cards indicating the random assignment and group allocation details were sealed in opaque envelopes by the same independent person. The physical therapist then opened these envelopes and administered the exercise program based on the assigned group.

### Assessments

For a schedule of data collection and measures list, refer to Table 1.

**Table 1:** Measures and schedule for data collection.

Measure name			Screener		Baseline		24-week follow-up
**Demographics information**							
	Age	✓					
	Gender	✓					
	Weight	✓					
	Height	✓					
	Marital status	✓					
	Alcohol consumption	✓					
	Duration of smoking (years)	✓					
	Occupation	✓					
	HLA-B27^a^	✓					
	ESR^b^	✓				✓	
**Past history or symptoms at onset**							
	Psoriasis	✓					
	IBD^c^	✓					
	Uveitis	✓					
	Hypertension	✓					
	Hip pain	✓					
	Low back pain	✓					
	Neck pain	✓					
	Upper back pain	✓					
	Hip joint pain	✓					
	Other joint pain	✓					
	Disease duration (months)	✓					
**Family history**							
	Hypertension	✓					
	Diabetes	✓					
	AS^d^	✓					
**Physical examination**							
	Heel pain	✓		✓		✓	
	Joint pain	✓		✓		✓	
	Anterior chest pain	✓		✓		✓	
	Sacroiliac joint pain	✓		✓		✓	
	Hip pain	✓		✓		✓	
	Chest expansion			✓		✓	
	Finger-to-floor distance			✓		✓	
	Five Times Sit to Stand Test			✓		✓	
	4-meter walk test			✓		✓	
	ASDAS-CRP^e^			✓		✓	
	ASAS HI^f^			✓		✓	
	BASDAI^g^			✓		✓	
	BASFI^h^			✓		✓	
	BASMI^i^			✓		✓	
	MASES^j^			✓		✓	
	HAMA-14^k^			✓		✓	
	HAMD-17^l^			✓		✓	
	VAS^m^ night pain			✓		✓	
	VAS 24-h pain			✓		✓	
	PGA^n^			✓		✓	
	PhGA^o^			✓		✓	
**SF-36^p^**							
	Physical function			✓		✓	
	Physiological role			✓		✓	
	Bodily pain			✓		✓	
	General health			✓		✓	
	Vitality			✓		✓	
	Social functioning			✓		✓	
	Emotional role			✓		✓	
	Mental health			✓		✓	
	Overall health			✓		✓	
	Adherence					✓	

^a^HLA-B27: human leukocyte antigen B27.

^b^CRP: C-reactive protein.

^c^IBD: inflammatory bowel disease.

^d^AS: ankylosing spondylitis.

^e^ASDAS-CRP: Ankylosing Spondylitis Disease Activity Score with C-reactive protein.

^f^ASAS HI: Assessment of Spondyloarthritis International Society Health Index.

^g^BASDAI: Bath Ankylosing Spondylitis Disease Activity Index.

^h^BASFI: Bath Ankylosing Spondylitis Functional Index.

^i^BASMI: Bath Ankylosing Spondylitis Metrology Index.

^j^MASES: Bath Ankylosing Spondylitis Functional Index.

^k^HAMD-17: 17-item Hamilton Depression Scale.

^l^HAMA-14: the 14-item Hamilton Anxiety Scale.

^m^VAS: visual analog scale.

^n^PGA: patient global assessment.

^o^PhGA: physician global assessment.

^p^SF-36: 36-Item Short Form Survey.

#### Primary Outcome

ASDAS, including total back pain, patient global assessment of disease activity, peripheral pain or swelling, duration of morning stiffness, and C-reactive protein level, is the primary outcome [10].

#### Secondary Outcomes

BASDAI, BASFI, and BASMI [11], which are used to reflect disease activity, will also be evaluated.

Assessment using the Spondyloarthritis International Society Health Index (ASAS HI) is performed to determine the common difficulties of patients with AS [12]. In addition, health-related quality of life is measured using the SF-36, including physical function; role of physical, bodily pain, general health, vitality, social functioning; and role of emotional, and mental health [13].

Hamilton Depression Scale (HAMD) is performed before and after the intervention. It is summarized into seven types of factor structures: (1) anxiety or somatization: mental anxiety, physical anxiety, gastrointestinal symptoms, hypochondriasis, and insight; (2) weight: weight loss; (3) cognitive disorders: guilt, suicide, agitation, depersonalization and derealization, paranoid symptoms, and obsessive‐compulsive symptoms; (4) day and night changes; (5) block: depression, work and interest, block and sexual symptoms; (6) sleep disorder: difficulty falling asleep, lack of sleep and early awakening; and (7) feeling of despair: feeling of loss, hopelessness and low self‐esteem. Scores of <8 points or less: no depression; 8 to <20 points: prone to depression; 20 to <35 points: depression; ≥35 points: severe depression.

Hamilton Anxiety Scale (HAMA) is performed before and after the intervention. Score interpretation is as follows: ≤7 points indicate no clinically significant anxiety; 8-14 points suggest possible (mild) anxiety; 15-21 points reflect moderate anxiety; and ≥ 22 points indicate severe anxiety.

Exercise adherence is defined as the ratio of the actual completed amount of exercise (recorded via the Healbone platform) to the recommended amount (3-4 sessions/wk), which will be quantified and compared between groups.

### Statistical Analyses

Statistical tests will be performed with SPSS Statistics (version 29; IBM Corp). Significance is defined as *P*<.05. Quantitative data are reported as means and SD. Statistical significance for quantitative variables is assessed using Student *t* test or 1-way ANOVA. Categorical data are presented as percentages, and significant differences are determined using chi-square tests.

### Power Calculations

The sample size was calculated using ASDAS-CRP as the primary outcome. Based on a previous randomized controlled trial comparing supervised versus home-based exercise in patients with AS, the study of the effect size was 0.714 [6]. Assuming a 2-tailed independent *t* test with α=.05, power 1−β=.8, and a 1:1 allocation ratio, G*Power 3.1 estimated a required sample size of 32 participants per group (total N=64). To account for a conservative 20% attrition rate-consistent with dropout rates reported in comparable AS exercise trial, we increased the total sample size to N=80 (40 per group).

### Missing Data

Missing data due to participant dropout will be addressed using the Last Observation Carried Forward (LOCF) method for the primary analysis. Under this approach, missing values at the 24-week follow-up will be replaced with the last available measurement from earlier timepoints. This method assumes that unobserved outcomes remain stable after dropout, a rationale consistent with prior studies in chronic conditions like AS, where short-term interventions may yield sustained effects. All randomized and consented patients, constituting the full analysis set, are going to be included according to intention-to-treat principles for the efficacy analyses.

### Ethical Considerations

This study was approved by the Ethics Committee of the Chinese PLA General Hospital (number S2022-738-02) in compliance with the Declaration of Helsinki. All participants provided written consent after being informed of the study’s goals, procedures, risks, and benefits. They retained the right to withdraw at any time without affecting their care. Participant data were anonymized and securely stored on the Healbone platform using encryption, two-factor authentication, and restricted access. Personal details (eg, names) were stored separately. No financial payment was provided. Participants received standard clinical care and exercise programs for AS. No identifiable images or personal information appeared in this paper. Any future inclusion of such content will require explicit written consent.

## Results

The recruitment of the first participant began on February 27, 2023. We have screened 216 outpatients who may be eligible subjects, among them 126 outpatients who initially met the inclusion criteria. After evaluating clinicians performed face-to-face assessments, 23 lacked a stable medication regimen, 5 presented with severe cervical vertebral bridges, 3 reported regular structured exercise, 2 could not be reached after prescreening, 10 had significant cardiovascular disease, and 3 declined participation due to time constraints. Ultimately, 80 eligible participants were enrolled, with 36 randomly allocated to the intervention group and 44 to the control group (Figure 1). Recruitment was completed on March 5, 2024, and the final participant was expected to complete the study in September 2024. Data analysis (November 2024) and result dissemination (April 2025) are pending.

## Discussion

### Principal Findings

This study anticipates that the digital functional exercise program (Healbone), which combines wearable sensors and real-time feedback, will lead to greater reductions in disease activity (ASDAS-CRP) and improvements in physical function (BASFI and BASMI) compared to standard home-based exercises. These benefits are expected to arise from higher adherence rates in the intervention group, driven by the platform’s reminders and corrective guidance. Existing research consistently shows that supervised exercise programs outperform home-based regimens in managing AS but face practical challenges like cost and accessibility [14-16]. Healbone bridges this gap by using wearable sensors and real-time feedback to mimic supervised guidance, addressing key limitations of traditional home-based methods (eg, poor adherence monitoring). This aligns with evidence that digital tools (eg, apps and wearables) improve exercise accuracy and compliance in chronic diseases. Exercise is known to reduce disease activity, pain, and stiffness in AS while enhancing physical function and quality of life [17]. However, its effectiveness depends on adherence, which is often low in home-based settings. Healbone tackles this.

Digital-powered remote system enables real-time quantitative assessment and precision-guided rehabilitation for patients with AS through sensor-based tracking, which can promote improvements in health and physical function. Studies indicate that the intensity, frequency, and duration of exercise intervention are crucial for its effectiveness [18]. However, previous studies have shown that the optimal mechanical load range for patients with spondyloarthritis is significantly narrower than that for normal individuals, proposing the concept of the “Goldilocks zone” for exercise load [19]. Supervised exercise rehabilitation guidance is constrained by time and location, which may affect long-term patient participation. In contrast, online exercise guidance platforms offer greater convenience and flexibility but may lack the ability to provide effective feedback, movement correction, and timely verbal encouragement [20]. Our system establishes a standardized exercise training model and provides real-time feedback and correction during exercise rehabilitation, effectively ensuring that patients engage in safe and effective exercise within their capabilities. This approach is expected to further enhance the clinical treatment outcomes for patients with AS and, expected to be widely implemented in outpatient rehabilitation settings.

### Limitations

This study has several limitations. One is that the patients are recruited for PLA General Hospital, which may limit the generalizability of the results to the rest of China and internationally. However, patients come from across the country, accordingly reducing some regional differences. Second, the differing monitoring strategies between groups-digital tracking for the intervention group versus self-reporting for the control group—may influence adherence comparisons. While this reflects real-world disparities between standard and technology-enhanced care, it introduces potential measurement bias. Future studies could use matched monitoring methods (eg, wearable devices for both groups) to isolate the effect of the digital platform itself, independent of adherence measurement tools. Finally, the exclusion of regularly active individuals may reduce the generalizability of our results. However, this approach aligns with prior trials aiming to evaluate novel interventions in controlled settings.

### Conclusions

To our knowledge, this is the first randomized controlled trial to assess a digital functional exercise program, enhanced with real-time feedback and sensor-based monitoring, for improving disease activity and physical function in patients with AS. If effective, this program could offer a scalable, cost-efficient solution for home-based rehabilitation, addressing the critical need for precise, supervised exercise in AS management. By leveraging current technologies, this approach fills a significant gap in chronic disease care, with potential applicability to other musculoskeletal conditions.

## Appendix

Multimedia Appendix 1 CONSORT (Consolidated Standards of Reporting Trials) 2010 checklist.

Multimedia Appendix 2 Example for a personalized workout program.

## Abbreviations

AS: ankylosing spondylitis
ASAS: Assessment of SpondyloArthritis international Society
ASAS HI: Assessment of Spondyloarthritis International Society Health Index
ASDAS-CRP: Ankylosing Spondylitis Disease Activity Score with C-reactive protein
BASDAI: Bath Ankylosing Spondylitis Disease Activity Index
BASFI: Bath Ankylosing Spondylitis Functional Index
BASMI: Bath Ankylosing Spondylitis Metrology Index
CONSORT: Consolidated Standards of Reporting Trials
DFEP: Digital Functional Exercise Program
EULAR: European League Against Rheumatism
GDPR: General Data Protection Regulation
HAMA: Hamilton Anxiety Scale
HAMD: Hamilton Depression Scale
LOCF: Last Observation Carried Forward
PLA: People’s Liberation Army
SF-36: 36-item Short Form Survey
VAS: visual analog scale

## Data Availability

The datasets generated or analyzed during this study are available from the corresponding author on reasonable request.
